# Structural alterations of the choroid evaluated using enhanced depth imaging optical coherence tomography in patients with coronavirus disease

**DOI:** 10.5935/0004-2749.20220066

**Published:** 2025-08-22

**Authors:** Özkan Kocamış, Emine Temel, Lokman Hızmalı, Nazife Aşıkgarip, Kemal Örnek, Fikriye Milletli Sezgin

**Affiliations:** 1 Department of Opthalmology, Kırşehir Ahi Evran University School of Medicine, Kırşehir, Turkey; 2 Department of Opthalmology, Kırşehir Ahi Evran Training and Research Hospital, Kırşehir, Turkey; 3 Department of Infectious Disease and Clinical Microbiology, Kırşehir Ahi Evran University School of Medicine, Kırşehir, Turkey; 4 Department of Medical Microbiology, Kırşehir Ahi Evran University School of Medicine, Kırşehir, Turkey

**Keywords:** Choroid, COVID-19, Coronavirus infections, tomography, optical coherence, Coróide, COVID-19, Infecções por coronavirus, Tomografia de coerência óptica

## Abstract

**Purpose:**

To assess choroidal changes using enhanced depth imaging optical coherence tomography in coronavirus disease (COVID-19).

**Methods:**

Thirty-two patients with moderate COVID-19 and 34 healthy subjects were included in the study. Choroidal thickness was measured at 3 points as follows: at the subfovea, 1500 mm nasal to the fovea, and 1500 mm temporal to the fovea. The total choroidal area, luminal area, stromal area, and choroidal vascular index were measured with Image-J. All the measurements were performed during the disease and at 4 months after remission.

**Results:**

In the patient group, the subfoveal, nasal, and temporal choroidal thicknesses were decreased as compared with those in the controls, but without statistically significant differences (p=0.534, p=0.437, and p=0.077, respectively). The mean total choroidal, stromal, and luminal areas and choroidal vascular index were statistically significantly decreased in the patient group (p<0.001, p=0.001, p=0.001, and p=0.003; respectively). At 4 months after remission, the choroidal structural parameters and choroidal vascular index revealed statistically significant increases as compared with the baseline measurements in the patients with COVID-19 (all p<0.001 and p=0.047, respectively).

**Conclusion:**

The choroidal vascular and stromal parameters showed significant transient decreases during the disease course of COVID-19.

## Abstract (pt)

**Objetivo:**

Avaliar alterações da coroide através de imagens de tomografia de coerência óptica (OCT) com pro fundidade realçada na doença por coronavírus de 2019 (COVID-19).

**Métodos:**

Foram incluídos no estudo 32 pacientes com COVID-19 moderada e 34 indivíduos saudáveis. A espessura da coroide foi medida em 3 pontos: subfoveal, a 1500 mm da fóvea na direção nasal e a 1500 mm da fóvea na direção temporal. A área total da coroide, a área luminal, a área estromal e o índice vascular da coroide foram medidos com o programa ImageJ. Todas as medições foram feitas durante a doença ativa e 4 meses após a remissão.

**Resultados:**

No grupo de pacientes, as espessuras subfoveal, nasal e temporal da coroide mostraram-se reduzidas em comparação com os controles. A diferença não foi estatisticamente significativa (respectivamente, p=0,534, p=0,437 e p=0,077). As médias das áreas total da coroide, estromal e luminal, bem como o índice vascular da coroide, mostraram-se diminuídos com significância estatística no grupo de pacientes (respectivamente, p<0,001, p=0,001, p=0,001 e p=0,003). Aos 4 meses após a remissão, os parâmetros estruturais e o índice vascular da coroide revelaram um aumento estatisticamente significativo em pacientes com COVID-19, em comparação com as medidas iniciais (todos os valores de p<0,001 para os parâmetros estruturais e p=0,047 para o índice vascular da coroide).

**Conclusão:**

Os parâmetros vasculares da coroide e do estroma mostraram uma diminuição transitória, mas significativa em pacientes com COVID-19 durante a doença.

## INTRODUCTION

Coronavirus disease (COVID-19) is extremely contagious, causing severe acute respiratory distress syndrome, and can lead to death especially in patients with concomitant systemic disease^(1)^. The disease has rapidly become widespread, resulting in an epidemic throughout China, followed by a pandemic, with an increasing number of cases in various countries worldwide^(2)^.

The exact pathophysiological mechanism of the COVID-19 infection remains largely unknown. However, it has been proposed that during the disease course, immune dysregulation and the high levels of proinflammatory cytokines could be the main cause of tissue injury^(3)^. In addition, some studies showed that vascular damage, thrombosis, and dysregulation of immune-mediated inflammation play important roles in the pathogenesis of severe COVID-19 infection^(4)^.

COVID-19 has been shown to affect different parts of the body. However, current studies on the ocular effects of COVID-19 are limited. As far as we know, ophthalmic changes are limited to external diseases such as conjunctivitis^(5-7)^. Furthermore, ocular manifestations such as retinitis, uveitis, and optic neuritis have been reported to occur owing to coronavirus infections in various animal models^(8)^. Casagrande et al. showed that viral ribonucleic acid is detectable in the retina of patients with COVID-19^(9)^.

Enhanced depth imaging optical coherence tomography (EDI-OCT) is a noninvasive imaging tool that enables visualization of retinal and choroidal structural alterations in numerous ocular and systemic conditions^(10,11)^. Considering that COVID-19 has been shown to cause vascular dysfunction and inflammation, we hypothesized that the disease may also affect the choroid and its stromal and vascular structures. Therefore, the aim of our study was to assess choroidal structural changes using EDI-OCT and Image-J in patients with COVID-19.

## METHODS

### Patient selection

This prospective cross-sectional study included patients who were hospitalized for confirmed COVID-19 at the Kırşehir Ahi Evran University Training and Research Hospital. A total of 32 COVID-19 patients (32 eyes, group 1) and 34 healty subjects (34 eyes, group 2) were included in the study. All the patients were positive for COVID-19 in real-time reverse transcriptase-polymerase chain reaction tests using nasopharyngeal swabs.

The study was performed in adherence to the tenets of the Declaration of Helsinki and was approved by the institutional review board of the Kırşehir Ahi Evran University School of Medicine (Decision number: 202010/79). Each patient was informed about the aims and methods of the study, and informed consent was obtained from all the patients.

All the patients had moderate COVID-19. The criteria for hospitalization were dyspnea (shortness of breath at rest), oxygen saturation <90%, and symptoms of hypoxia (mental confusion, coughing, severe headache, and tachycardia) with abnormal laboratory findings. None of the patients in the study were admitted to the intensive care unit. The treatment protocol included oral hydroxychloroquine and favipiravir for 5 days and subcutaneous enoxaparin sodium for 1 month. None of the patients had any other blood flow examinations such as color Doppler imaging during hospitalization.

The right eye of each patient was included in the study. The exclusion criteria were as follows: retinal diseases such as retinal vascular occlusive disease, hypertensive retinopathy, diabetic retinopathy, central serous chorioretinopathy, age-related macular degeneration, degenerative myopia, intraocular pressure >22 mmHg, any systemic abnormalities (e.g., vascular disease, hypertension, or diabetes mellitus), history of previous intraocular surgery or laser photocoagulation, smoking, several types of systemic medication, and caffeine intake. Patients with poor-quality EDI-OCT images due to corneal or lens opacities were also excluded from the study.

### Imaging and image analysis

Slit-lamp biomicroscopy and EDI-OCT (Spectralis, Heidelberg Engineering Inc., Heidelberg, Germany) measurements were performed by the ophthalmologists while wearing full protective equipment in group 1. The same measurements were also performed for the eyes included in the control group.

**Figure 1:**
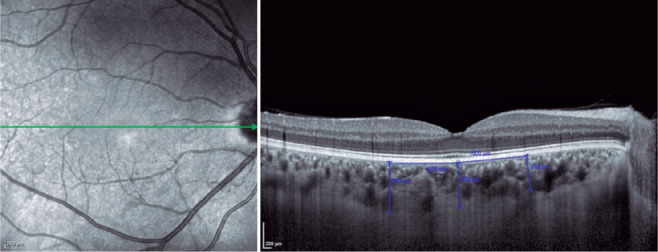
Choroidal thickness measurements at 3 points as follows: at the subfovea, 1500 µm nasal to the fovea, and 1500 µm temporal to the fovea.

Choroidal thickness was measured from the outer portion of the hyperreflective line, corresponding to the retinal pigment epithelium, to the inner surface of the sclera. The choroidal thickness measurements were made at the following 3 points: at the subfovea, 1500 µm nasal to the fovea, and 1500 µm temporal to the fovea (Figure 1). EDI-OCT images were recorded at the same time of the day (9:00 am to 12:00 pm) to avoid the influence of diurnal variations. All the measurements were performed by 2 independent masked observers at baseline and 4 months after remission.

**Figure 2:**
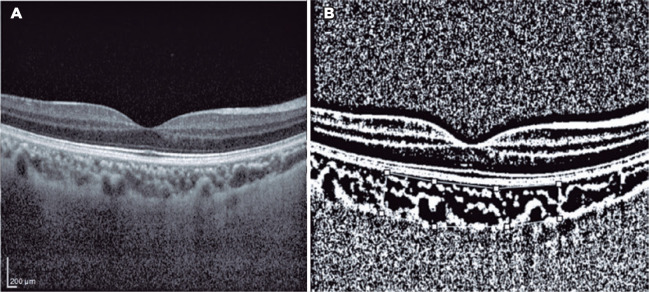
(A) Enhanced depth imaging optical coherence tomography image of the eye of a healthy subject. (B) Converted binary image using Image-J, with the area of interest in the choroid demarcated with a white line. The choroidal area was measured at approximately 3000 µm wide, with margins of 1500 µm nasal and 1500 µm temporal to the foveal center. The light pixels were evaluated as the stromal area; and the dark pixels, as the luminal area.

**Figure 3:**
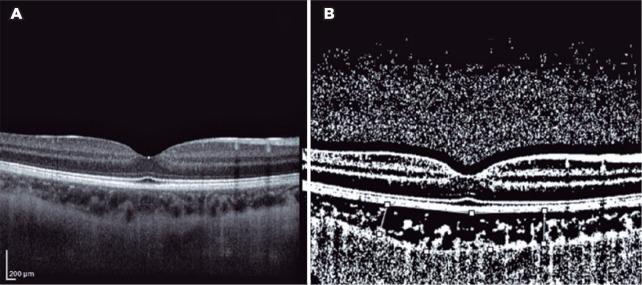
(A) Enhanced depth imaging optical coherence tomography image of the eye of a patient with COVID-19 infection. (B) Converted binary image using Image-J, with the area of interest in the choroid demarcated with a white line. The choroidal area was measured at approximately 3000 µm wide, with margins of 1500 µm nasal and 1500 µm temporal to the foveal center. The light pixels were evaluated as the stromal area; and the dark pixels, as the luminal area.

Binarization of the choroidal area was performed with the Image-J Version 1.50a software (National Institutes of Health, Bethesda, MD, USA; Figures 2 and 3).

The EDI-OCT image was opened with Image-J, and 3000-µm wide areas with margins of 1500 µm temporal to the fovea was chosen. The choroidal area was defined as the region from the retinal pigment epithelium to the chorioscleral border, and the borders were set manually with the Image-J ROI Manager. Three choroidal vessels with lumens >100 µm were selected using the oval selection tool and the mean reflectivity of the luminal areas were determined. The Niblack method was used for the binarization of the choroidal image. Then, the image was converted to 8 bits and adjusted using the Niblack auto local threshold. The luminal area was determined with the threshold tool. After adding the distance between the pixels, the choroidal, luminal, and stromal areas were automatically calculated for the 2 groups. The light pixels were accepted as the stromal area; and the dark pixels, as the luminal area ^(12)^. The choroidal vascularity index (CVI) was calculated as the ratio between the luminal and total choroidal areas.

### Statistical analyses

All the comparisons between the 2 groups were statistically analyzed using SPSS 11.5 (SPSS Inc., Chicago, IL, USA). The normality of all the data was tested with the Kolmogorov-Smirnov test. The significance of the differences between the groups was investigated using one-way analysis of variance and the Kruskal-Wallis test. Pairwise comparisons with the Tukey HSD and Bonferroni tests were used to evaluate which group had significant differences. Intraclass correlation coefficients were used for the assessment of the reliability of all the measurements. Statistical significance was set at p<0.05.

## RESULTS

Of the patients with COVID-19, 14 (43.7%) were female, and the mean age was 35.9 ± 21.6 years (range: 8-87 years). Twenty (58.8%) of the patients in the control group were female, and the mean age was 37.2 ± 14.9 years (range: 10-63 years). No statistically significant differences were found between the 2 groups in terms of age and sex (p=0.667 and p=0.452, respectively). Table 1 shows the demographic and clinical data of the patients with COVID-19 and healthy subjects.

**Table 1:** Demographic and clinical data of the patients with COVID-19 (group 1) and healthy subjects (group 2)

	Group 1	Group 2
Patients, n (%)	32 (48.5)	34 (51.5)
Eyes, n (%)	32 (48.5)	34 (51.5)
Female, n (%)	14 (43.7)	20 (58.8)
Male, n (%)	18 (56.2)	14 (41.2)
Age (years), mean±SD (range)	35.9 ± 21.6 (8-87)	37.2 ± 14.9 (10-63)

Of the patients with COVID-19, 7 (21.9%) had abnormal findings on computed chest tomography. Two patients (28.6%) with abnormal chest tomography findings had symptoms such as cough and shortness of breath. In addition, among the patients with COVID-19, 5 (15.6%) had abnormal laboratory findings such as elevated C-reactive protein level and reduced number of lymphocytes. No drug-related adverse effects were observed in the patients with COVID-19 during the treatment and follow-ups.

None of the patients, both symptomatic/asymptomatic and those with abnormal chest tomography findings, had coexisting ocular symptoms or findings. No abnormalities were found on the ocular surface and in the anterior chamber or posterior segment on slit-lamp examination.

**Figure 4:**
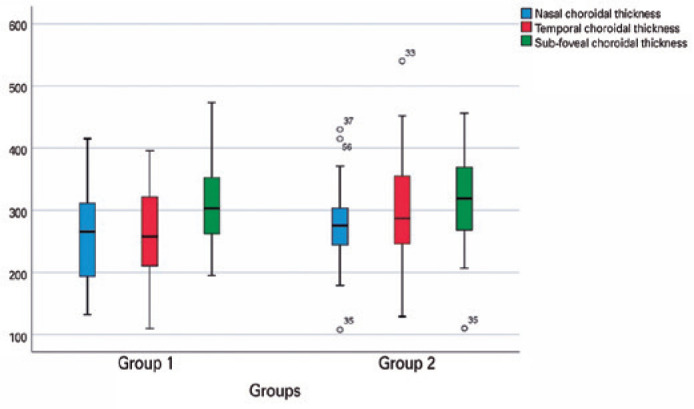
The distribution of choroidal thickness changes detected on the enhanced depth imaging optical coherence tomography images of the patients with COVID-19 (group 1) and healthy subjects (group 2).

The mean subfoveal choroidal thickness was 311.21 ± 74.10 µm in the patient group and 322.91 ± 77.56 µm in the controls. The mean choroidal thickness at 1500 µm nasal to the fovea was 260.31 ± 80.62 µm in the patient group and 274.29 ± 64.21 µm in the controls. The mean choroidal thickness at 1500 µm temporal to the fovea was 261.71 ± 74.27 µm in the patient group and 297.73 ± 87.57 µm in the controls. In the patients, the subfoveal, 1500-µm nasal, and 1500-µm temporal choroidal thicknesses were decreased as compared with those in the healthy subjects. No statistically significant differences were found between the 2 groups (p=0.534, p=0.437, and p=0.077, respectively). The distribution of choroidal thickness changes among groups is shown in figure 4.

According to the Image-J data, the mean total choroidal area was 0.540 ± 0.17 mm^2^ in the patient group and 0.957 ± 0.21 mm^2^ in the controls, with a statistically significant difference (p<0.001). The mean stromal area was 0.164 ± 0.05 mm^2^ in the patient group and 0.229 ± 0.08 mm^2^ in the controls. The mean stromal area was decreased statistically significantly in the patients with COVID-19 (p=0.001). The mean luminal area was 0.376 ± 0.15 mm^2^ in the patient group and 0.727 ± 0.16 mm^2^ in the controls. When compared, the values showed a statistically significant difference (p=0.001).

**Figure 5:**
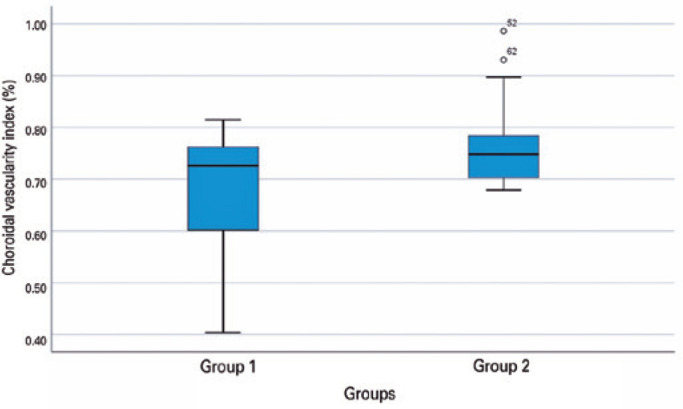
Distribution of CVI changes detected using binarization of the enhanced depth imaging optical coherence tomography images of patients with COVID-19 (group 1) and healthy subjects (group 2).

The mean CVI was 67.91 ± 6.2 in the patient group and 76.11 ± 8.1 in the control group. The mean CVI was statistically significantly decreased in the patient group as compared with the healthy subjects (p=0.003). The distribution of CVI changes between the groups is shown in figure 5. Table 2 lists the choroidal structural characteristics of the patients with COVID-19 and healthy subjects.

**Table 2:** Choroidal structural characteristics of the patients with COVID-19 (group 1) and healthy subjects (group 2)

Variable (Mean ± SD)	Group 1	Group 2	p value
Subfoveal choroidal thickness (µm)	311.21 ± 74.10	322.91 ± 77.56	0.534
Choroidal thickness at 1500 µm nasal to the fovea (µm)	260.31 ± 80.62	274.29 ± 64.21	0.437
Choroidal thickness at 1500 µm temporal to the fovea (µm)	261.71 ± 74.27	297.73 ± 87.57	0.077
Total choroidal area (mm^2^)	0.540 ± 0.17	0.957 ± 0.21	<0.001^*^
Stromal area (mm^2^)	0.164 ± 0.05	0.229 ± 0.08	0.001^*^
Luminal area (mm^2^)	0.376 ± 0.15	0.727 ± 0.16	0.001^*^
CVI (%)	67.91 ± 6.2	76.11 ± 8.1	0.003^*^

SD= Standard deviation; CVI= Choroidal vascularity index.

* = Statistically significant p values.

At 4 months after remission, the CVI and choroidal structural parameters showed statistically significant increases as compared with the baseline values (p=0.047 and all p<0.001, respectively; Table 3), but no statistically significant differences were found between the patient and control groups (all p>0.05) (Table 4). The intraclass correlation coefficient and confidence intervals of all the measurements are listed in table 5.

**Table 3:** Comparison of the choroidal structural characteristics at baseline and after the remission period in the patients with COVID-19

		Mean±SD		
	**Baseline**	**Remission**		**p value**
Subfoveal choroidal thickness (µm)	311.21 ± 74.10	316.18 ± 64.12		0.662
Choroidal thickness at 1500 µm nasal to the fovea (µm)	260.31 ± 80.62		268.24 ± 78.21	0.554
Choroidal thickness at 1500 µm temporal to the fovea (µm)	261.71 ± 74.27		279.32 ± 69.43	0.332
Total choroidal area (mm^2^)	0.540 ± 0.17		0.881 ± 0.086	<0.001^*^
Stromal area (mm^2^)	0.164 ± 0.05		0.223 ± 0.07	<0.001^*^
Luminal area (mm^2^)	0.376 ± 0.15		0.658 ± 0.113	<0.001^*^
CVI (%)	67.91 ± 6.2		74.84 ± 8.7	0.047^*^

SD= Standart deviation; CVI= Choroidal vascularity index.

* = Statistically significant p value.

**Table 4:** Comparison of the choroidal structural characteristics after the remission period between the patients with COVID-19 and the controls

	Mean ± SD		
	Patients (Remission)	Controls	p value
Subfoveal choroidal thickness (µm) 316.18 ± 64.12 322.91 ± 77.56 0.523			
Choroidal thickness at 1500 µm nasal to the fovea (µm)	268.24 ± 78.21	274.29 ± 64.21	0.498
Choroidal thickness at 1500 µm temporal to the fovea (µm)	279.32 ± 69.43	297.73 ± 87.57	0.232
Total choroidal area (mm^2^)	0.881 ± 0.086	0.957 ± 0.21	0.065
Stromal area (mm^2^)	0.223 ± 0.07	0.229 ± 0.08	0.756
Luminal area (mm^2^)	0.658 ± 0.113	0.727 ± 0.16	0.061
CVI (%)	74.84 ± 8.7	76.11 ± 8.1	0.385

SD= Standard deviation; CVI= Choroidal vascularity index.

* = Statistically significant p value.

**Table 5:** Intraclass correlation coefficient and confidence intervals of all the measurements

	Cronbach’s alpha coefficient	95% CI	
		Lower	Upper
Subfoveal choroidal thickness (µm)	0.996	0.992	0.998
Choroidal thickness at 1500 µm nasal to the fovea (µm)	0.995	0.992	0.997
Choroidal thickness at 1500 µm temporal to the fovea (µm)	0.996	0.993	0.998
Total choroidal area (mm^2^)	0.997	0.993	0.998
Stromal area (mm^2^)	0.972	0.942	0.986
Luminal area (mm^2^)	0.998	0.998	0.999
CVI (%)	0.997	0.994	0.999

Cl= Confidence interval; CVI= Choroidal vascularity index.

## DİSCUSSİON

Coronavirus has been shown to manifest in different parts of the human body, including the gastrointestinal system and eye, besides the respiratory system^(13,14)^. Most clinical studies about coronavirus have focused on the respiratory system because of its life-threatening nature. Evaluation of other organ systems should be taken into consideration, as it may help to provide valuable information for uncovering the obscure mechanisms of tissue injury.

Ocular involvement in patients with COVID-19 is limited to the conjunctiva and tear film layer, as reported in previous studies^(5,6,15)^.”” Viral ribonucleic acid can be detected in the retina of people with the infection^(9)^. In a study by Seah et al., coronaviruses were shown to produce various ocular manifestations ranging from anterior segment pathologies such as conjunctivitis and anterior uveitis to vision-threatening conditions such as retinitis and optic neuritis^(8)^.

The factors that trigger severe disease in individuals with COVID-19 infection are not completely understood. Hyperinflammation and coagulopathy have been shown to contribute to disease severity and death in patients with COVID-19^(16)^. In addition, clinical studies suggest that severe COVID-19 infection reflects a confluence of vascular dysfunction and disruption in thrombotic mechanisms^(4)^.

The choroid is the vascular part of the eye and plays an important role in the pathogenesis of several ocular diseases. The choroidal circulation is a dense network of capillaries located behind the retinal pigment epithelial cell layer. The choriocapillaris, which forms the innermost layer of the choroid, is the fundamental blood supply to the outer retina. Owing to the high metabolic demand of the photoreceptor layer, the choroid receives most of the blood (65-85%) supplied to the retinal structures^(17)^.

Impaired choroidal blood flow is associated with several ocular diseases such as glaucoma, retinitis pigmentosa, degenerative myopia, and age-related macular degeneration. Histological analysis has revealed that changes in the choroidal interstitial stroma may occur in eyes with age-related macular degeneration due to edema, fibrosis, and inflammation with cellular infiltration^(18)^.

In the present study, we compared the luminal and stromal areas of the choroid in patients with COVID-19 and healthy subjects by using the binarization technique with Image-J. Our findings showed that the mean total choroidal, luminal, and stromal areas were statistically significantly decreased in the patients with COVID-19 as compared with the healthy subjects.

Choroidal thickness is a parameter that varies substantially in both healthy and pathological conditions. It decreases from the macula to the periphery and is at its maximum subfoveally. Choroidal thickness does not provide a true representation of the entire choroidal vasculature as an objective marker. Measuring CVI provides information about the vascular and stromal components of the choroid. Xin et al. reported that CVI was independent from systemic and ocular factors such as age, axial length, intraocular pressure, or systolic blood pressure^(19)^. However, choroidal thickness may vary depending on these factors. In a study by Xin et al., the mean CVI reported for healthy subjects was 70.12%^(19)^. In our study, this ratio was 76.11% in the controls. Agarwal et al. found in their study that the subfoveal CVI was 65.61% by including 345 eyes of healthy subjects with a mean age of 61 years^(20)^. In our study, we found a CVI of 76.11%, but the mean age of our control group was 37 years. The normative CVI value in healthy people was studied, and age-related changes were evaluated for CVI^(21,22)^. Jaeryung et al. showed no significant correlations between CVI and age^(21)^. By contrast, Ruiz-Medrano et al. reported that CVI was significantly higher in subjects aged 18 years than in a group of older people^(22)^.

Some studies have reported regarding CVI and its potential applications in the evaluation, diagnosis, and treatment of diseases of the retina and choroid, such as central serous chorioretinopathy, polypoidal choroidal vasculopathy, panuveitis, and diabetic retinopathy^(23-27)^. Agrawal et al. assessed CVI in people with posterior uveitis and panuveitis and showed an increased CVI in the affected eye, which had significantly decreased at 3-month follow-up^(25)^. Significant choroidal changes such as decreased CVI were also reported in eyes with serpiginous choroiditis associated with tuberculosis^(27)^. Shulin et al. demonstrated a lower CVI during the active period of the Vogt-Koyanagi-Harada disease and hypothesized that decreased CVI during the active period was due to choroidal stromal edema and infiltration by inflammatory cells^(28)^.

In our study, we observed that the mean CVI was decreased in the patients with COVID-19 infection as compared with healthy subjects. According to our hypothesis, vascular damage, hypercoagulability, and hyperinflammation factors, which have been shown to be involved in the pathogenesis of COVID-19 infection, might have led to ischemia of the choriocapillaris and decreased CVI. In addition, the reduction in the luminal and stromal areas and CVI measured with Image-J might be explained by a secondary consequence of decreased oxygen demand. At 4 months after remission, the values all the choroidal parameters were increased close to the levels of the control group.

We also compared choroidal thickness changes between the patient and control groups. The subfoveal, 1500-µm nasal, and 1500-µm temporal choroidal thicknesses were decreased in the patients with COVID-19. Although the choroidal thickness was decreased at all points, as did the CVI in the patient group, we could not find a statistically significant difference.

The limitations of our study include the relatively small sample size and absence of detailed ocular examinations due to the logistic challenges of managing patients with COVID-19. Binarization of choroidal images was performed only in the right eye of each participant; therefore, inter-eye differences might have affected the results.

In conclusion, this study is the first to show that a significant transient decrease in choroidal vascular and structural parameters can occur during the acute phase of COVID-19. Further studies with a larger sample size are needed to clarify the choroidal structural and vascular changes and determine the ocular blood flow changes in patients with COVID-19.
